# Functional Properties of Brewer’s Spent Grain Protein Isolate: The Missing Piece in the Plant Protein Portfolio

**DOI:** 10.3390/foods12040798

**Published:** 2023-02-13

**Authors:** Alice Jaeger, Aylin W. Sahin, Laura Nyhan, Emanuele Zannini, Elke K. Arendt

**Affiliations:** 1 School of Food and Nutritional Science, University College Cork, T12K8AF Cork, Ireland; 2 Department of Environmental Biology, Sapienza University of Rome, Piazzale Aldo Moro 5, 00185 Rome, Italy; 3 APC Microbiome Institute, University College Cork, T12YT20 Cork, Ireland

**Keywords:** brewers’ spent grain, sustainability, brewing by-products, protein functionality, valorisation

## Abstract

Plant protein sources, as a part of developing sustainable food systems, are currently of interest globally. Brewer’s spent grain (BSG) is the most plentiful by-product of the brewing industry, representing ~85% of the total side streams produced. Although nutritionally dense, there are very few methods of upcycling these materials. High in protein, BSG can serve as an ideal raw material for protein isolate production. This study details the nutritional and functional characteristics of BSG protein isolate, EverPro, and compares these with the technological performance of the current gold standard plant protein isolates, pea and soy. The compositional characteristics are determined, including amino acid analysis, protein solubility, and protein profile among others. Related physical properties are determined, including foaming characteristics, emulsifying properties, zeta potential, surface hydrophobicity, and rheological properties. Regarding nutrition, EverPro meets or exceeds the requirement of each essential amino acid per g protein, with the exception of lysine, while pea and soy are deficient in methionine and cysteine. EverPro has a similar protein content to the pea and soy isolates, but far exceeds them in terms of protein solubility, with a protein solubility of ~100% compared to 22% and 52% for pea and soy isolates, respectively. This increased solubility, in turn, affects other functional properties; EverPro displays the highest foaming capacity and exhibits low sedimentation activity, while also possessing minimal gelation properties and low emulsion stabilising activity when compared to pea and soy isolates. This study outlines the functional and nutritional properties of EverPro, a brewer’s spent grain protein, in comparison to commercial plant protein isolates, indicating the potential for the inclusion of new, sustainable plant-based protein sources in human nutrition, in particular dairy alternative applications.

## 1. Introduction

Protein ingredients isolated from plant sources are gaining popularity, in part due to the increasing emphasis by the consumer on health and ethics, as well as the worldwide movement towards sustainability and reducing the impact on the environment [1,2]. Popular protein ingredients currently on the market include pea, soy, rice and oat, among some other legumes and grains. However, in recent years with the concept of sustainability and upcycling gaining increased traction, the extraction and valorisation of protein ingredients from food processing side streams are of particular interest.

Alongside this, the demand for plant-based dairy alternatives is on the increase. However, in many instances, the nutritional value of these products is sub-par when compared to their animal-based counterparts. In particular, plant-based milk substitutes are nutritionally lacking with regard to protein. A study by Jeske et al. (2017) evaluated commercially available plant-based milk alternatives and found that 50% of the analysed products had a low protein content, with 11 out of 17 containing <1% protein, compared to bovine milk (~3.5%). Only beverages prepared with soy protein could be described as comparable to bovine milk with regards to protein content [3], which cannot be consumed by everyone due to its allergy potential. With the increasing interest of the consumer in dairy alternative products, an expansive arsenal of plant-based protein ingredients with a wide range of functionalities is vital to produce nutritionally comparable dairy alternative products.

Barley (*Hordeum vulgare, vulgare L*) is one of the most widely cultivated crops and is the main grain used in the production of alcoholic beverages worldwide [4]. Barley represents the majority of the grain remnants after completion of the brewing process, known as brewer’s spent grain (BSG). This is the most voluminous waste product produced by the brewing industry, representing ~85% of the total by-products produced [5]. As part of the ongoing global effort to minimise food processing waste and increase upcycling of by-products, the potential extraction and valorisation of proteins from BSG on a large scale is an expanding area of research. Spent barley grain is rich in protein, containing approx. 19–30% protein [6,7,8]. Of this, approx. 30% consists of essential amino acids [8]. The main proteins in barley are prolamins (or hordeins) and glutelin, which both act as storage proteins within the barley seed. During malting and mashing, these proteins are broken down into smaller peptides of free amino acids, mainly via enzymatic degradation [9,10]. While the functionality of innate barley proteins has been documented, studies examining the functional characteristics of barley proteins extracted from BSG are scarce.

In contrast to this, pea (*pisum sativum L.*) is one of the most commonly used and well-researched plant protein sources available to consumers and to food businesses looking to include plant proteins in their products. Several different brands are offering pea protein isolates and many products are formulated using pea protein ingredients, including milk alternatives, dried snacks, bars, meat alternatives, among many other food and beverage products. Pea protein is known for its high nutritional value, especially regarding amino acids composition, low allergenicity, and widespread availability, as well as its low cost [11,12]. The main protein constituents of the pea seed are the salt-soluble globulins (70–80%) and the water-soluble albumins (10–20%). The globulins can further be broken down into legumin and vicilin with minor amounts of a third sub-protein known as convicilin [12,13]. 

Similarly to pea, soybean (*glycine max*) is another widely researched and commonly used source of plant-based protein, and has been in use since ancient times [14]. Soy is recognised for its high nutritional value, containing all essential amino acids, and possessing good functional characteristics. This has led to its use in a wide range of products, similar to pea protein ingredients. Soybeans contain approx. 40% protein by dry matter and the main components are glycinin and β-conglycinin, making up more than 80% of total soy proteins [15].

The nutritional and functional characteristics of both pea and soy proteins have been widely documented, whereas the functional properties of BSG protein, as compared to more commonly used plant protein sources, remains largely unknown. While barley is typically the grain primarily associated with brewing, and therefore BSG, it is also common that adjuncts are utilised as supplemental carbohydrate sources during the brewing process [16,17]. In the case of EverPro, rice was used in addition to barley. Therefore, EverPro is a concentration of BSG proteins from these two sources.

This research article aims to characterise this unique source of protein and determine its ability to replicate, and potentially even exceed, those functional characteristics displayed by well-known and widely accepted plant proteins. Characteristics such as solubility, foaming behaviour and gelling abilities, will be strategic for the implementation of EverPro, and other alternative proteins, as ingredients in a variety of food products for human consumption. 

## 2. Materials and Methods

### 2.1. Materials

EverPro Barley-Rice protein isolate was obtained from EverGrain Ingredients (St. Louis, MO, USA). Pea protein isolate (PPI) and soy protein isolate (SPI) were obtained from Naturz Organics (Helmond, Netherlands). All chemicals were sourced from Sigma–Aldrich (St. Louis, MO, USA), unless otherwise stated.

### 2.2. Compositional Analysis

Compositional analysis was carried out using the following methods: protein was measured using the Kjeldahl method (AACC Method 46-12) [18] using a nitrogen-to-protein conversion factor of 6.25; fat content was quantified using the AACC Method 30-25.01 [19]; moisture was determined using the oven-drying method (AACC Method 44-15.02) [20]; total starch was determined using the Megazyme kit K-RAPRS (Bray, Ireland); sugar content was determined by HPLC, using the extraction method of Hoehnel et al. (2020) [21]. Sugars were quantified by HPLC on an Infinity 1260 system with a refractive index detector (Agilent Technologies, Palo Alto, CA, USA), using a Sugar-Pak I column (300 mm × 6.5 mm; Waters Corporation, Taunton, MA, USA), and an eluent of 0.0001 M CaEDTA at a flow rate of 0.5 mL/min and a column temperature of 80 °C. Maltotriose, sucrose, lactose, glucose, fructose and mannitol were used as external standards. The amino acid composition was determined externally by Chelab S.r.l. (Resana, Italy), using ion chromatography with post-column derivatisation with ninhydrin, or HPLC-UV analysis in the case of tryptophan.

### 2.3. Quantification of Fermentable Oligo-, Di-, Mono-Saccharides and Polyols (FODMAPs)

The quantification of mono-, di-, galactooligosaccharides, fructans, and polyols were conducted using high-performance anion-exchange chromatography coupled with pulsed amperometric detection (HPAEC-PAD), performed on a DionexTM ICS-5000+ system (Sunnyvale, CA, USA), as described by Ispiryan et al. (2019) [22]. All carbohydrates, except for the fructans, were quantified using authentic reference standards. The total fructan content was determined after enzymatic hydrolysis with two enzyme mixtures, A (containing α-galactosidase and amyloglucosidase) and B (Megazyme, Bray, Ireland). Enzyme mixture B contained the same enzymes as A, along with fructan-degrading inulinases. The total fructan content was calculated based on the content of free (A) and released (B) glucose, fructose and sucrose as described in [22]. All levels below 0.025 g/100 g are not detected (n.d.). All extractions were carried out in triplicate. The results are presented as gram analyte per 100 g sample on a dry weight basis (g/100 g DM).

### 2.4. pH and Total Titratable Acidity (TTA)

The pH and TTA were determined using the method of Waters et al. [23], with modifications as described by Neylon et al. [24]. Specifically, 10 g of the sample was weighed into a beaker, and 95 mL of distilled water and 5 mL of acetone were added. Then, samples were mixed using a stirring bar until well dispersed. The pH was measured using a pH meter (Mettler Toledo, Columbus, OH, USA) and the TTA was determined by titration with 0.1 M NaOH until a pH of 8.5 was reached. Following this, a 3 min waiting time took place. If the pH changed at this time, the titration and waiting time steps were repeated until the pH was steady. The TTA is expressed as mL 0.1 M NaOH.

### 2.5. Foaming Capacity and Stability

Dispersions with a sample concentration of 2% (*w/v*) were prepared using distilled water. The pH was adjusted to pH 7 using HCl and NaOH (Sigma–Aldrich/Fisher Scientific, St. Louis, MO, USA) of varying concentrations and the samples were hydrated overnight at 4 °C. After equilibrating to room temperature and further pH adjustment, if necessary, the samples were frothed using an Ultra-Turrax equipped with a S10N-10G dispersing element (IKA Labortechnik, Janke and Kunkel GmbH, Staufen, Germany), at maximum speed for 30 s. The height of the sample (foam phase only) was measured immediately and after 60 min. Foaming capacity was measured as % sample expansion at 0 min, while foam stability was measured as the sample expansion at 60 min as a percentage of sample expansion at 0 min. Sample expansion was calculated using the following equations [25]:

(1) $$Foaming\;capacity\;\left(\%\right)=\left(\frac{Foam\;height\;immediately\;after\;foaming}{Initial\;sample\;height}\right)\times 100$$

(2) $$Foam\;stability\;\left(\%\right)=\left(\frac{Foam\;height\;after\;1\;hour}{Foam\;height\;immediately\;after\;foaming}\right)\times 100$$

### 2.6. Fat Absorption Capacity

To determine fat absorption capacity (FAC), 1 g of sample powder and 6 g of sunflower oil were weighed into a 15 mL tube, dispersed using a vortex mixer (Daihan Scientific, Seoul, Republic of Korea) for 3 min at the highest speed and centrifuged at 4000× *g* for 30 min. The oil was decanted from the tube carefully and the pellet was re-weighed. The FAC (%) was determined using the following equation [26]:

(3) $$FAC\left(\%\right)=\frac{\left(Weight\;of\;tube+pellet\right)-\left(Weight\;of\;empty\;tube\right)}{Weight\;of\;ingredient}\times \frac{100}{1}$$

### 2.7. Protein Profile Analysis

An Agilent Bioanalyzer 2100 lab-on-a-chip capillary electrophoresis system (Agilent Technologies, United States) was used to analyse the protein profile and estimate the molecular weights of the respective protein bands. Samples were prepared as described in Vogelsang et al. [26], with some slight modifications. Protein ingredients were dispersed in 2% SDS, 2 M thiourea and 6 M urea to obtain a protein concentration of 2 mg/mL. Dispersions were shaken for 2 h at 22 °C and centrifuged to remove insoluble material. Samples were analysed using an Agilent Protein 80 kit and Protein 230 kit (St. Louis, MO, USA) according to the instructions within the ranges of 5–80 and 14–230 kDa, respectively. The Protein 80 kit was used for EverPro due to increased levels of smaller peptides. For reducing conditions, dithiothreitol (DTT) was included.

### 2.8. Particle Size

Particle size distribution was measured using a static laser light diffraction unit (Mastersizer 3000, Malvern Instruments Ltd., Worcestershire, UK) [25,26,27], covering a size range of 0.01–3000 µm. Particle size was analysed on both a dry and wet basis. Wet samples were prepared as described by Vogelsang et al. [25], 1% (*w/v*) protein dispersions were prepared using ultrapure water in 50 mL centrifuge tubes, adjusted to pH 7 using HCl and NaOH of varying concentrations, and shaken at 500 rpm overnight at 4 °C to hydrate. The samples were then allowed to equilibrate to 22 °C. The particle refractive index was set to 1.45 and the dispersant refractive index was set to 1.33. Sample dispersions were introduced into the dispersing unit using ultrapure water as a dispersant, until a laser obscuration of 12% (PPI and SPI) and 5% (EverPro) was achieved. For measurement on a dry basis, the same refractive parameters were used.

### 2.9. Minimum Gelation Concentration

The minimum gelation concentration of each protein was determined as described by Vogelsang et al. [25], with slight adjustments. Dispersions (15 mL) of varying concentrations (6–22%) were prepared in 50 mL centrifuge tubes using distilled water. The samples were then adjusted to pH 7 using varying concentrations (0.01 M–2 M) of HCl and NaOH, and hydrated overnight at 4 °C. Samples were heated at 90 °C in a water bath for 30 min, cooled rapidly on ice, and maintained overnight at 4 °C. The samples were then inverted, and the minimum protein concentration at which the dispersion did not flow in less than 30 s was determined as the minimum gelling concentration.

### 2.10. Rheological Characteristics

Rheological tests were carried out using a controlled stress rheometer (MCR301, Anton Paar GmbH, Vienna, Austria) equipped with a concentric cylinder measuring system (C-CC27-T200/SS, Anton Paar GmbH, Vienna, Austria), as described by Vogelsang et al. [25]. Ingredient dispersions, based on minimum gelation concentrations, were hydrated overnight at 4 °C. Following this, the samples were adjusted to room temperature, sheared for 20 s at speed 1 with an Ultra-Turrax T10 equipped with a S10N-10G dispersing element (IKA Labortechnik, Janke and Kunkel GmbH, Staufen, Germany), and pH was then adjusted to 7.0. Since no minimum gelation concentration was determined for EverPro, EverPro was analysed at concentrations of 14% and 8%, the minimum gelation concentrations of PPI and SPI, respectively. Small deformation oscillatory rheology was used to monitor heat gelation with a constant strain and frequency of 0.1% and 1 Hz, respectively. The temperature profile used was as follows: the temperature was increased from 20 to 90 °C at 2 °C/min, held at 90 °C for 30 min, cooled to 20 °C at 2 °C/min, and held at 20 °C for 30 min. This was followed by a logarithmic frequency sweep from 0.01 to 10 Hz, and a constant strain at 1%.

### 2.11. Surface Hydrophobicity

The surface hydrophobicity was measured using the method described by Vogelsang et al. [28]. Protein dispersions were serially diluted with 10 mM phosphate buffer (pH 7) in the range of 0.0006–0.015% (*w/v*). Then, 8-Anilino-1-naphthalenesulfonic acid ammonium salt (ANS)(10 µL; 8.0 mM in 0.1 M phosphate buffer, pH 7) was mixed with 2 mL of diluted sample and stored in the dark for 15 min at room temperature. Fluorescence was measured (λ _excitation_ 390 nm, λ _emission_ 470 nm) and corrected using blanks, sample dilutions measured without the addition of ANS. The results are presented as the slopes (R^2^ ≥ 0.98) of the absorbance versus protein concentration (% *w/v*).

### 2.12. Protein Solubility

For evaluation of protein solubility, dispersions of 1% (*w/v*) protein were prepared, and the pH was adjusted to pH 7 using HCl or NaOH. Dispersions were hydrated at 4 °C, shaking overnight. The temperatures of the samples were then readjusted to 22 °C while shaking, followed by a readjustment of the pH if necessary. Samples were centrifuged at 4893× *g* for 30 min and the protein contents of the resultant supernatants were measured using the Kjeldahl method (N × 6.25). Protein solubility was expressed as the % of protein remaining in the supernatant [28].

### 2.13. Zeta Potential

The zeta potential of the ingredients in solution was determined using dynamic light scattering technology with the Zetasizer Nano-Z (Malvern Instruments Ltd., Worcestershire, UK) according to the method described by Vogelsang et al. (2022) [28]. Samples (0.1% *w/v* protein) were prepared using ultrapure water and adjusted to pH 7 using varying concentrations of HCl or NaOH. Samples were incubated by shaking at 4 °C overnight. Samples were then readjusted to room temperature and centrifuged at 2000× *g* for 10 min to remove any insoluble material. Automatic voltage selection was used for measurements and the zeta potential was calculated using the Smoluchowski model. A refractive index of 1.45 and an absorbance of 0.001 were used.

### 2.14. Emulsifying Characteristics

Emulsion stability was examined using the method described by Vogelsang et al. (2021) [29], with some adjustments. Aqueous sample dispersions of 1.2% *w/v* concentration were prepared, adjusted to pH 7 using varying concentrations of HCl and NaOH, and hydrated by shaking overnight at 4 °C. Emulsions were prepared by mixing ingredient dispersions with sunflower oil with a ratio of 90:10 (material dispersion: oil) in 50 mL centrifuge tubes. Samples were sheared using an Ultra-Turrax equipped with a S10N-10G dispersing element (IKA Labortechnik, Janke and Kunkel GmbH, Staufen, Germany) at speed 5 for 2 min. Oil droplet size was measured using a static laser light diffraction unit (Mastersizer 3000, Malvern Instruments Ltd., Worcestershire, UK) with a refractive index of 1.47 and 1.33 for sunflower oil and water, respectively. Stability was monitored using an analytical centrifuge (LUMiSizer, LUM GmbH, Berlin, Germany) with parameters of 100 rcf for 15 min at 15 °C. The results are reported as separation rate (%/min) and transmission profiles over the entire measurement range.

### 2.15. Colour

The sample colour was measured using the hand-held Minolta colour measuring system (Chroma meter CR-400/410, Konica Minolta, Tokyo, Japan) [24,30], with adaptations for dry powder ingredients. The powders were loaded into flat glass petri dishes and a flat surface was created. Five measurements were taken per dish and each dish was emptied, refilled and remeasured in triplicate. Colour was measured using the CIE colour system (XYZ values), and then translated into and reported using the Hunter colour system (L*a*b*).

### 2.16. Ultrastructure

Scanning electron microscopy (SEM) was used to image the ultrastructure of PPI, SPI and EverPro, according to the method reported by Atzler et al. [31]. Ingredients were mounted on stubs (G 306; 10 mm × 10 mm Diameter; Agar Scientific, Essex, UK) and fixated using carbon tape (G3357N; Carbon Tabs 9 mm; Agar Scientific, Essex, UK). The samples were then sputter coated with a gold–palladium alloy (ratio of 80/20), using a Polaron E5150 sputter coating unit, and imaging was executed with a JEOL scanning electron microscope (JSM-5510, Jeol Ltd., Tokyo, Japan). The following settings were applied for the analysis: 5 kV voltage, 20 mm working distance and a magnification factor of 50 and 1000.

### 2.17. Statistical Analysis

All experiments were performed in triplicate. Data was analysed at a 5% level of significance using a one-way ANOVA, followed by Tukey’s post-hoc test in IBM SPSS version 26 (Armonk, NY, USA).

## 3. Results

### 3.1. Nutritional Characteristics

The nutritional characteristics of a protein isolate are key when considering the ideal protein ingredients for food application. 

Compositional Analysis

The composition of the PPI, SPI and EverPro are presented in Table 1. The moisture values for all three samples are similar, although a statistical difference does exist between them. As expected, all three isolates have high protein contents, with soy having the highest at 89 g/100 g DM, followed by pea and EverPro, which contained 81 and 83 g/100 g DM, respectively. Lipid content is low in EverPro and SPI (<1.75 g/100 g DM), whereas PPI has a significantly higher lipid content of 8.5 g/100 g DM. All isolates contained low levels of sugars (0.06–0.30 g/100 g DM), and digestible starches are also low, with levels ranging between 1 and 3 g/100 g DM. The PPI contained the highest level of FODMAPs (0.53 g/100 g DM), followed by SPI (0.04 g/100 g DM). However, the FODMAP value of EverPro was below the level of quantification and is therefore represented as not detected (n.d.).

### 3.2. Protein Characteristics

#### 3.2.1. Amino Acid Composition

The amino acid compositions, free amino acids and the percentage of the recommended essential amino acid content per gram of protein are presented in Table 2. EverPro contains significantly more glutamic acid and proline than both PPI and SPI. However, there is less arginine (4.7 g/100 g DM) and lysine (3.04 g/100 g DM) in EverPro than in PPI and SPI. Regarding the nutritional characteristics, all three ingredients contain high levels of the majority of essential amino acids. However, both PPI and SPI are lacking in the sulphur-containing amino acids (<1 g/100 g DM), particularly cysteine, while EverPro (2.77 g/100 g DM) reaches the recommended level as outlined by the World Health Organisation [32]. The only instance where EverPro fails to meet the requirement is with lysine. EverPro contains only 3.04 g/100 g DM of lysine, while PPI and SPI contain 6.4 and 5.3 g/100 g DM, respectively. No free amino acids were detected in SPI, while only low levels of free arginine, glutamine and leucine were detected in PPI. In contrast to this, EverPro contained a variety of free amino acids, with the highest levels being seen for glutamic acid (0.168 ± 0.028 g/100 g DM), phenylalanine (0.132 ± 0.022 g/100 g DM), leucine (0.217 ± 0.036 g/100 g DM) and tyrosine (0.108 ± 0.019 g/100 g DM). The total free amino acids for EverPro were 1.25 g/100 g, compared to only 0.08 g/100 g in PPI and non-detectable levels in SPI.

#### 3.2.2. Protein Profiles

**Figure 1:**
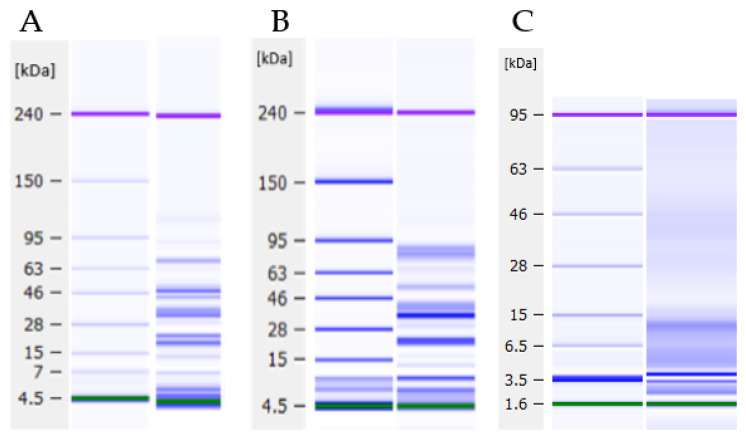
Protein profiles for pea (**A**), soy (**B**) and EverPro (**C**), as analysed using the Bioanalyzer under reducing conditions.

The protein profiles of the three ingredients were analysed using the Bioanalyzer lab-on-a-chip technology under reducing and non-reducing conditions. Due to minimal observable differences, only reducing conditions are shown in Figure 1. Both PPI and SPI display bands that can be identified from the literature, including convicilin, vicilin and legumin in pea, and β-conglycinin and glycinin in soy. The bands at ~65 kDa and ~48 kDa most likely represent the albumins convicilin and vicilin, respectively. Similarly, the dark band at ~20 kDa is assumed to be the corresponding basic subunits. Regarding EverPro, there is evidence of small peptides, indicated by increased colour density between ~15 kDa and ~4 kDa. There is also a slight indication of peptides between 46 kDa and 28 kDa. The lack of defined bands, as opposed to those that can be seen in PPI and SPI, is to be expected as the small peptides are the result of protein degradation, producing peptides of varying molecular weights. However, the Bioanalyzer has a cut-off of 3.5 kDa, therefore, the presence of peptides below this level cannot be identified.

### 3.3. Physical Properties

#### 3.3.1. pH and Total Titratable Acidity

The pH and total titratable acidity (TTA) are displayed in Table 3. All of the pH values are within the neutral range, with EverPro having the highest pH at pH 7.90, followed by SPI at pH 6.77, and PPI at pH 6.36. EverPro displayed the highest TTA of 17.8 mL 0.1 M NaOH, while PPI and SPI have similar TTA values of 11.4 mL and 12.1 mL, respectively. Statistically significant differences can be observed between the pH values, while the TTA values for pea and soy are not statistically different.

#### 3.3.2. Protein Solubility, Surface hydrophobicity and Zeta Potential

The protein solubility, surface hydrophobicity and zeta potential of PPI, SPI, and EverPro are presented in Table 3. A statistically significant difference in solubility can be observed, with EverPro being the most soluble ingredient at 101.71% ± 2.9 (expressed as a percentage of total protein), followed by SPI at 51.96% ± 3.4, and PPI at 22.27% ± 1.5. In terms of surface hydrophobicity, SPI exhibited the highest hydrophobicity with 7471 a.u. (arbitrary units), while the result for PPI was 4292 a.u. It was not possible to determine the surface hydrophobicity of EverPro, likely due to the increased amounts of small peptides as a result of protein degradation. The zeta potential of SPI (−33.8 mV) and EverPro (−30.0 mV) at pH 7 are not statistically different from each other, but are significantly higher than that of PPI (−22.6 mV).

#### 3.3.3. Colour

**Figure 2:**
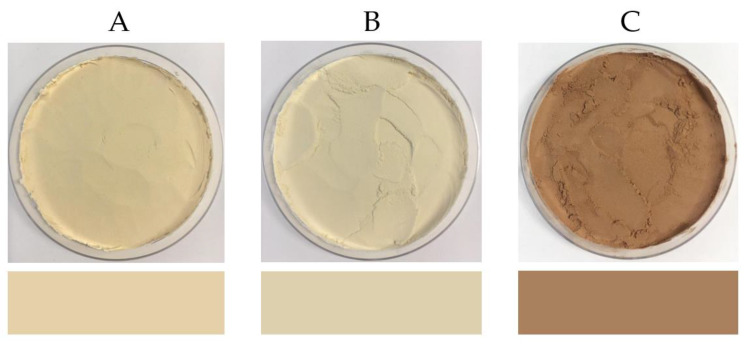
Images of PPI (**A**), SPI (**B**), and EverPro (**C**), alongside a digital visualisation of the L*a*b* values for each sample.

The colour values and images of PPI, SPI and EverPro are shown in Table 3 and Figure 2, respectively. The L*a*b* colour values for PPI and SPI are relatively similar, with PPI having a slightly higher a* value, indicating an increased red tone. The b* values were also similar, ranging from 18.5 ± 0.13 for SPI to 24.98 ± 0.80 for EverPro. The EverPro sample has a much darker colour, having a statistically significant lower L* value of 57.4 compared to 84.7 and 83.8 for PPI and SPI, respectively, which do not differ significantly.

#### 3.3.4. Particle Size

**Figure 3:**
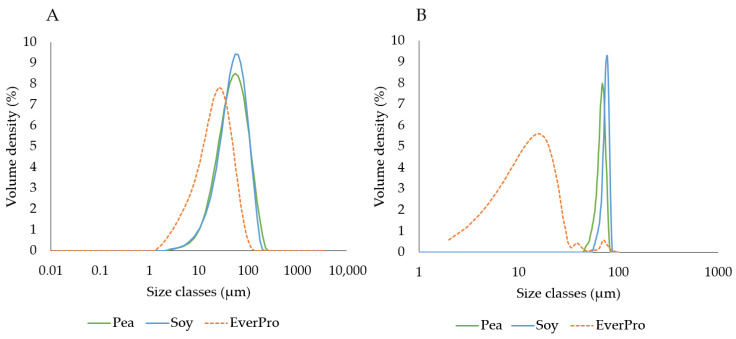
The dry (**A**) and wet (**B**) particle size dispersion for pea, soy and EverPro, displayed as volume density (%) per size class (μm).

The dry (A) and dispersed (B) particle sizes of PPI, SPI, and EverPro are shown in Figure 3. Regarding the dry powders, EverPro has the smallest particle size with an average D[4,3] of 24.67 ± 0.058 μm, while PPI and SPI are very similar, with average D[4,3] of 58.63 ± 0.058 μm and 56.57 ± 1.069 μm, respectively. In dispersion, EverPro has a much smaller particle size than PPI and SPI, with a D[4,3] mean of 12.22 ± 5.05 µm compared to 70.48 ± 2.59 µm and 144.33 ± 8.65 µm, respectively. Standard deviation for EverPro is high due to its near total solubility, which is not optimal for this type of analysis. In the case of EverPro, the D[3,2] average may be a more accurate measure of particle size in dispersion, due to its increased sensitivity to smaller particles. The D[3,2] values for PPI, SPI and EverPro are 32.22 ± 1.15 µm, 88.17 ± 6.09 µm, and 0.05 ± 0.00 µm, respectively.

#### 3.3.5. Fat holding Capacity

Fat absorption capacity is a characteristic that can be related to emulsifying capacity, and is therefore, important for consideration in fat-rich applications, such as sauces and dairy products. As shown in Table 3, EverPro displayed the highest fat absorption capacity of 182.35% ± 1.91, followed by PPI and SPI with 157.72% ± 3.20 and 120.05% ± 17.84, respectively. 

#### 3.3.6. Foaming Capacity and Stability 

**Figure 4:**
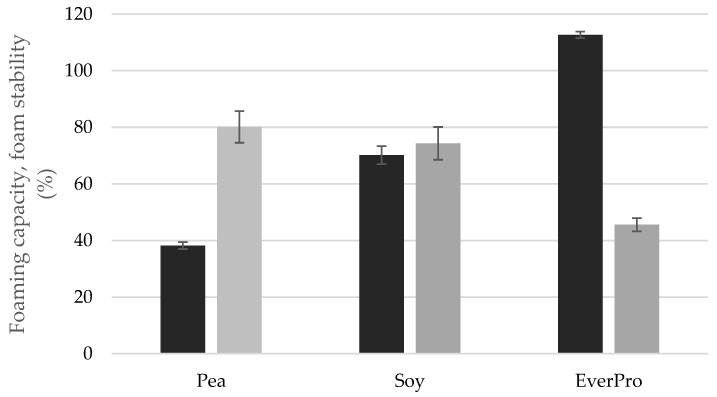
The foaming capacity (black bars) and foam stability (grey bars) of pea, soy and EverPro.

The foaming capacity and stability are displayed in Figure 4. EverPro had the highest foaming capacity with a value of 112.68% ± 1.16, followed by soy and pea with foaming capacities of 70.14% ± 3.18 and 38.19% ± 1.20, respectively. Foam height at 60 min after initial frothing showed poor foam stability in the case of EverPro (45.57% ± 2.23). Interestingly, although PPI had the poorest foaming capacity, it produced the most stable foam (80.12% ± 5.57), while SPI also displayed significant foam stability (74.31% ± 5.97). 

#### 3.3.7. Gelation Characteristics and Rheological Properties

**Figure 5:**
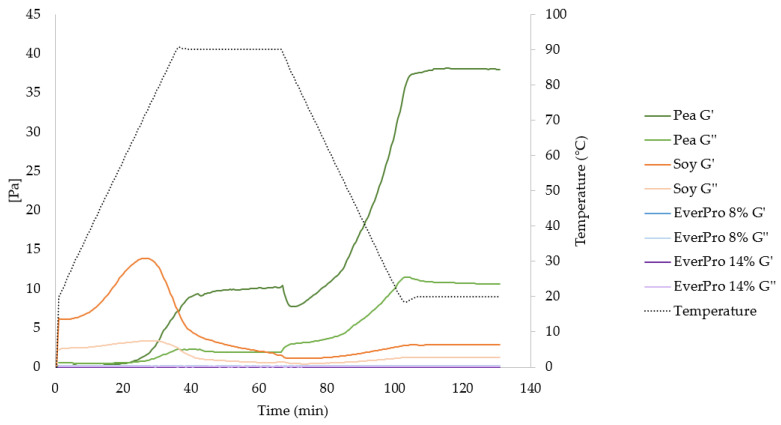
Rheological profiles of pea, soy and EverPro over a heating and cooling cycle, represented by the storage modulus (G’) and the loss modulus (G”).

The minimum gelation concentration for PPI and SPI were 14% and 8%, respectively. EverPro was trialled in concentrations of up to 22%, but gelling activity was not observed. During rheological analysis (Figure 5), PPI displays the strongest gelling behaviour initiating at the beginning of the heating stage and increasing greatly during cooling. Regarding SPI, G’ (storage modulus) also dominates over G” (loss modulus), with a much higher starting G’ than PPI, indicating gel formation before heating. This is also observed through an initial tan δ value of 0.36, as opposed to 2.99 for PPI. Upon the initiation of heating, G’ increases, but then dramatically decreases during the 90 °C hold, below the initial G’, indicating a structural breakdown under high heat treatment. In comparison to PPI and SPI, EverPro displays no gelling properties over a heating curve, as the G’ is equal to 0 throughout the measurement. Therefore, the tan δ approaches infinity at the attempt to divide G” by 0. A frequency sweep was also performed. For EverPro, at high frequencies G” dominates at both 8% and 14% levels. The opposite is true for PPI, while SPI exhibits a change from G’ dominant behaviour at low frequencies to G” dominant behaviour at higher frequencies.

#### 3.3.8. Emulsifying Characteristics

**Figure 6:**
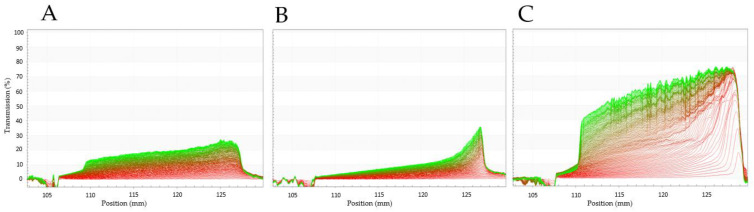
Light transmission profiles of pea (**A**), soy (**B**) and EverPro (**C**), where the left-hand side of the curves represents the top of the measurement cell.

The separation profiles of PPI, SPI and EverPro oil-in-water emulsions are shown in Figure 6. EverPro (C) separated most quickly, as evidenced by the significantly increased separation rate and the almost immediate increase in light transmission throughout the length of the cell and the formation of a cream layer, visible on the left side of the profile. In contrast, the emulsion prepared with the SPI dispersion showed the slowest phase separation, with a gradual increase in transmission beginning towards the bottom of the cell. There is also significant evidence of sedimentation, visible on the right side of the profile. The performance of PPI was between that of EverPro and SPI, with a gradual increase in light transmission, as well as the formation of a cream layer and a sediment layer. There were no significant differences in the D[3,2] (surface area moment mean) between the samples, with values of 8.77 μm, 10.16 μm, and 9.97 μm determined for PPI, SPI and EverPro, respectively. The D[4,3] (volume moment mean) values were also similar for all samples, ranging between 22.78 µm and 27.74 µm.

#### 3.3.9. Ultrastructure

**Figure 7:**
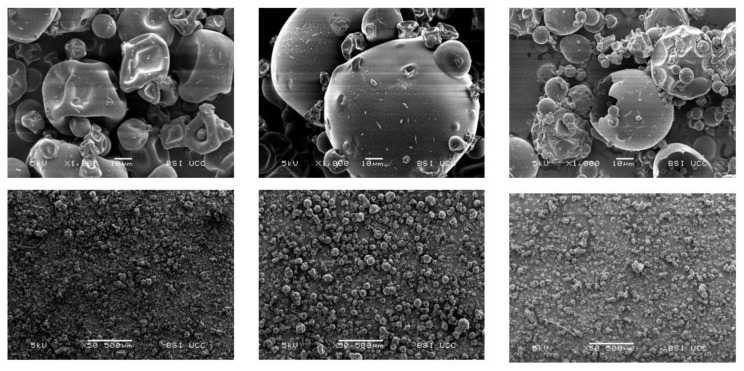
SEM images of PPI (**left**), SPI (**middle**), and EverPro (**right**) at ×1000 (top row) and ×50 (**bottom** row) magnification.

The ultrastructure of PPI, SPI and EverPro was examined using scanning electron microscopy (SEM), at 50× and 1000× levels of magnification (Figure 7). EverPro displays rounder particles, with a wide variety in particle size. There is also clear evidence of damaged particles. Alternatively, PPI and SPI display generally globular particles with dimpled surfaces. A range of different particles sizes can be observed in both.

## 4. Discussion

The characterisation of novel protein ingredients, such as EverPro, is key to their introduction as new protein sources for their application in day-to-day products. A wide range of functionality, as well as a high content of essential amino acids, allow competition with currently used protein sources like pea and soy.

The ingredient composition of the three samples is similar, with PPI, SPI, and EverPro containing 81%, 89% and 83% of protein, respectively. The only significant difference was observed in the fat content, whereby PPI contains significantly higher levels of lipids than SPI or EverPro. This relatively high lipid content could cause oxidation issues during storage, resulting in the formation of off-flavours [33]. In contrast, EverPro contains only trace amounts of fat, potentially due to the lower overall fat content in the grain, along with the impact of the extraction method used. Levels of FODMAPs also varied significantly between the samples, with none detected in EverPro. According to the literature, barley grains contain 0.9% to 4.2% fructans on a dry matter basis [34], as well as 0.56 g/100 g of galactooligosaccharides (GOS) [35], with the range due to, among other variables, differences in cultivars and growth conditions. Due to this, FODMAP content is reduced as a result of the transformation of barley grain to spent grain protein isolate. A study by Ispiryan et al. showed a slight increase of fructans in barley as a result of the malting process [36], indicating that any fructan degradation likely occurs after this point. It can be hypothesised that total FODMAPs are reduced as a result of enzymatic activity, particularly of endogenous α-galactosidases in malted grain [36],‘cooking’ during the mashing phase of the brewing process, or during the protein isolate extraction process [37]. A slightly higher level of FODMAPs in PPI is expected, as pulses are known to contain significant levels of GOS [35]. In a recent study, another commercially available pea protein isolate was found to contain 1.16 g/100 g DM GOS [35]. In comparison to this, PPI had a very low FODMAP content, likely due to differences in cultivar or production processes. Alkaline extraction followed by iso-electric point precipitation is the primary method of protein extraction from legumes [13], and is likely the method used to produce PPI and SPI, although the exact parameters are unknown. However, this would involve various solubilisation steps where soluble carbohydrates, including some FODMAPs, would have been removed [35].

Analysis of the total amino acid composition showed that EverPro has quite a similar profile to PPI and SPI, except for elevated levels of glutamine and proline. This is in agreement with a study conducted by Connolly et al., in which glutamine and proline were also found to be the most abundant amino acids in BSG protein extracts [38]. In terms of nutrition, all three protein ingredients perform well. However, both pea and soy are lacking in sulphur-containing amino acids, in particular cysteine. While EverPro does not have this issue, it fails to meet the requirement for lysine, although it does reach 81% of the total required value. EverPro also contained a much higher amount of free amino acids than PPI and SPI; it can be theorised that this is due to increased protein degradation during the malting, mashing and extraction processes [39]. It is widely known that a degree of protein degradation occurs during the brewing process. Malting, a controlled germination of the barley grain, leads to the activation of hydrolytic enzymes and initiates partial degradation of storage proteins in the grain [10,39,40]. It is estimated that up to 70% of the proteins are degraded to smaller peptides and amino acids by endoproteases, during this stage [10]. Further protein degradation occurs during the mashing step, due to the residual activity of the endogenous barley enzymes, which survive the kilning process. Also, it is common that additional proteases are added during this step to maximise free amino acid content, required for yeast metabolism. A combination of these processes is likely responsible for the protein degradation evident in EverPro. It is also possible that some protein degradation is occurring during the protein extraction process. Evidence of this protein degradation is also supported by the protein profiles, with a higher density of small peptides of all molecular weights visible with EverPro. Although the production processes for PPI and SPI are unknown, it appears that the isolates are in their native state and have not been subjected to protein degradation.

Legumin, an albumin found in many pulses, is made up of six subunit pairs, engaged in non-covalent bonds. Each of these pairs consists of an acidic subunit of ~40 kDa and a basic subunit of ~20 kDa [41], which can clearly be identified on the protein profile. Soy generally consists of glycinin (11S) and β-conglycinin (7S). Similar to pea legumin, soy glycinin is made up of three pairs of subunits, each bonded by a single disulphide bond. The acidic subunits can be clearly identified by a dark band at ~40 kDa, which is in agreement with the values given by [42,43]. Conglycinin, the second major storage protein of soy, is comprised of three subunits: α, α’, and β. These subunits can be seen represented by dark bands at ~70 kDa, ~80 kDa and ~50 kDa respectively, which is in agreement with the values seen in the literature [44].

Increased volumes of small peptides and amino acids are the primary reason behind the increased solubility of EverPro, compared to PPI and SPI. Protein solubility has a direct effect on many important functional properties, including foaming characteristics and emulsion-forming capabilities, due to changing surfactant activity as a result of conformational changes [45]. This can be seen with regards to foaming capacity, with EverPro having the highest foaming capacity while being the most soluble, while PPI had the lowest solubility and subsequently, a decreased foaming capacity. However, these trends were reversed with regard to foam stability. The increased solubility of EverPro is also responsible for the reduced particle size and minimal sedimentation effects observable in dispersion. While these are beneficial traits, its low emulsifying capability would require the addition of an emulsifier in a beverage application. This very low emulsifying activity is likely due to protein degradation, resulting in the loss of some surface functionality [46]. This is supported by a study conducted by Celus et al. (2007), where extensive protein hydrolysis of a BSG protein concentrate showed a reduction in emulsifying capacities and an improvement in foaming properties [47]. However, it could be of interest to explore the emulsifying capacity further, using a variety of different concentrations and mixing conditions.

Surface hydrophobicity (S_0_) is a quantification of the hydrophobic region on the surface of a protein molecule in a polar aqueous solution. Generally, proteins have a limited amount of these groups externally and this is increased by protein denaturation and unfolding, therefore exposing formerly buried hydrophobic groups. Following this, in theory, protein denaturation and degradation should increase S_0_. However, it is also known that excessive BSG protein degradation can cause a reduction in S_0_, simply due to a smaller number of hydrophobic regions being present in smaller peptides [47]. This appears to be the case with EverPro, where the S_0_ appeared to be non-existent to the point of being unmeasurable. Generally, S_0_ can also be positively correlated with emulsifying characteristics, with SPI displaying the slowest separation rate, followed by PPI and EverPro, respectively. Further to this, increased protein solubility can indicate increased emulsifying abilities [48], which can be observed in PPI and SPI. However, the opposite is true of EverPro, which has high solubility and low emulsifying capacity. Again, this indicates an extensive protein degradation has taken place during the production of EverPro, resulting in reduced surfactant properties [46].

Zeta potential is a measure of the charge, in mV, at the slipping plane of a particle in solution. This value can give an indication of the behaviour of the particle in solution, particularly with regard to solubility and aggregation. Regarding PPI, SPI and EverPro, zeta potential is relatively similar for all three protein ingredients, with values ranging between −23 mV to −34 mV. Generally, a zeta potential exceeding −30 mV or +30 mV is considered ‘stable’ [49]. Therefore, SPI and EverPro just reach this limit, while PPI falls short. The zeta values for PPI and SPI at pH 7 are comparable to those found in the literature [12,50,51]. In theory, zeta potential can be linked to protein solubility, with a zeta potential closer to zero (the iso-electric point) resulting in minimal solubility. It can be observed that PPI, with the lowest zeta potential value, is also the least soluble. Although, SPI and EverPro display similar zeta potentials, the high degree of protein degradation in EverPro explains the higher degree of solubility. Also, it is important to note that proteins are complex molecules and the measurement of protein surface properties, such as zeta potential and surface hydrophobicity, are highly sensitive to changes in pH, ionic strength, degree of hydrolysis, preparation method [52], and protein conformational changes during processing.

In addition to foaming properties and emulsifying characteristics, the gelling behaviour of protein ingredients is of great importance when considering potential applications and processing conditions. The results show that EverPro displays no gelling characteristics at concentrations up to 22% *w/w*, while PPI and SPI formed gels at 14% (*w/w*) and 8% (*w/w*), respectively. The gelling properties of pea proteins are well studied, with pea proteins generally forming a strong gel network upon heating, although this depends on certain parameters, such as pH, ionic strength and heating temperature, as well as cultivar, protein fraction ratios and protein extraction method [53,54,55,56]. A study by Sun & Arntfield (2010), determined a similar gelling concentration of 14.5% for a commercial pea protein isolate [55], while Shand et al. (2007) reported a value of 16% [56]. Regarding SPI, similar values to those obtained in this study have also been reported in the literature [57]. During heating, the storage modulus (G’) dominated over the loss modulus (G”) for PPI and SPI, indicating increased elastic behaviour and gel formation. Concerning SPI, G’ starts off relatively high, showing the formation of a cold-set gel before heating, represented by a tan δ value below 1 (0.36) [58]. However, the 90 °C hold causes a dramatic drop in G’, representing a reduction in the elastic properties of the material. This could be due to excessive denaturation of SPI at high temperatures, reducing the thickness of the dispersion. The domination of G” at higher frequencies also indicates a more viscous structure. The opposite is true of PPI, where gelling only begins upon heating and strengthens during cooling. This is also reflected in the tan δ values, where tan δ drops below 1 at 22 min. G’ dominates over G” throughout all tested frequencies, indicating the formation of a stronger network. It is important to note that gelling characteristics rely heavily on parameters such as pH, concentration, and temperature, among others. However, it may be theorised that the lack of gel formation with EverPro is a result of extensive protein degradation during malting, mashing, or further processing during extraction.

EverPro has a much darker colour than PPI or SPI. This could be due to a variety of factors, including the browning reaction occurring during the brewing process, as well as during the protein extraction process. Colour is produced, via the Maillard reaction, during the malting process as a result of kilning [59]. The degree of colour production can be manipulated by variations in temperature intensity and time [59]. Protein isolate colour is also affected by extraction processes, particularly pH [60].

For PPI and SPI, the ultrastructure images reflect what has been described and seen previously in the literature for these isolates, namely a variety in particle size with a generally spherical particle with a dimpled surface [61,62,63]. Regarding EverPro, there is clear evidence of damaged particles, likely a result of the processing methods. The extreme variety in particle size may be due to the presence of both barley and rice proteins. However, there is little to no comparable data available in the literature.

## 5. Conclusions

The increased consumption of novel plant-based proteins in the human diet is gaining momentum globally. In addition to this, the current climate crisis is driving interest in developing sustainable food systems. This study examined the properties of EverPro, a barley-rice protein isolated from brewer’s spent grain, in comparison to pea and soy protein isolates, and specifically aligns with the goals associated with SDG 12, Responsible Consumption and Production. Given its high solubility and high essential amino acid content, EverPro can be considered a highly functional protein ingredient suitable for use in a wide variety of food applications. EverPro exhibits a significant degree of protein degradation through a lack of visible bands during protein profile analysis, as along with increased levels of free amino acids. This perhaps makes it more similar in behaviour to a protein hydrolysate, instead of a typical protein isolate. This allows for some benefits, such as the near complete solubility, but means the ingredient lacks other functional properties, such as gelling and other network-forming capabilities. This property in particular highlights the potential for the application of EverPro in dairy-alternative beverages, where protein content and amino acid balance have shown a need to be improved upon.

The first protein of its kind, EverPro offers a new opportunity for further study to enrich a wide variety of plant-based products with high-quality protein, while also representing the result of a bio-circular food process; a future food system approach.

## Figures and Tables

**Table 1:** Compositional analysis of PPI, SPI and EverPro. Values in the same row with the same lowercase letter are not significantly different from each other.

	PPI	SPI	EverPro
	g/100 g DM		
Moisture	7.29 ± 0.11 ^a^	6.62 ± 0.08 ^b^	6.10 ± 0.05 ^c^
Protein	81.22 ± 0.43 ^a^	89.22 ± 0.33 ^b^	83.22 ± 1.11 ^c^
Fat	8.51 ± 0.07 ^a^	1.72 ± 0.17 ^b^	0.37 ± 0.06 ^c^
Total Sugars (Sucrose, Glucose, Fructose, Maltose)	0.19 ± 0.00 ^a^	0.06 ± 0.00 ^b^	0.30 ± 0.00 ^c^
Total Starch (digestible)	2.83 ± 0.06 ^a^	1.67 ± 0.03 ^b^	1.42 ± 0.04 ^c^
FODMAPs (Total)	0.53 ± 0 ^a^	0.04 ± 0 ^b^	n.d.*^c^

* n.d.: not detected or below the limit of quantification (LoQ) (<0.025%).

**Table 2:** Complete amino acid analysis of PPI, SPI and EverPro, comprising of total amino acids quantification on a dry matter basis, free amino acid quantification on a dry matter basis, and the determination of the % of the daily requirement of each essential amino acid per g of protein as outlined by the WHO (2007).

Essential Amino Acids	PPI		SPI		EverPro	
		% Requirement *		% Requirement *		% Requirement *
Histidine	2.227 ± 0.311	182.8	2.255 ± 0.314	168.5	1.768 ± 0.181	141.7
Isoleucine	3.955 ± 0.550	162.3	3.116 ± 0.434	116.4	2.673 ± 0.266	107.1
Leucine	7.422 ± 1.032	154.9	6.689 ± 0.931	127.1	6.145 ± 0.618	125.2
Lysine	6.399 ± 0.891	175.1	5.343 ± 0.743	133.1	3.035 ± 0.277	81.1
Methionine + cysteine	0.605 ± 0.075	33.9	0.942 ± 0.118	48.0	2.769 ± 0.296	151.3
Phenylalanine + tyrosine **	8.456 ± 0.843	273.9	8.16 ± 0.814	240.8	8.317 ± 0.597	263.1
Threonine	3.137 ± 0.437	167.9	3.281 ± 0.456	159.9	3.45 ± 0.351	180.3
Tryptophan	0.398 ± 0.043	81.7	0.690 ± 0.075	128.8	1.171 ± 0.128	234.7
Valine	4.433 ± 0.617	139.9	3.358 ± 0.468	96.5	4.004 ± 0.405	123.4
**Non-essential amino acids**						
Alanine	3.66 ± 0.509		3.571 ± 0.497		4.228 ± 0.426	
Arginine	8.422 ± 1.172		7.327 ± 1.019		4.707 ± 0.469	
Aspartic acid	9.286 ± 1.292		8.963 ± 1.248		7.699 ± 0.767	
Glutamic acid	14.549 ± 2.025		15.555 ± 2.164		20.234 ± 2.023	
Proline	4.035 ± 0.562		4.511 ± 0.628		8.019 ± 0.799	
Serine	4.694 ± 0.654		4.907 ± 0.683		4.164 ± 0.415	
Glycine	3.842 ± 0.535		3.688 ± 0.514		3.663 ± 0.373	
**Free Amino Acids**						
Glutamic acid	0		0		0.168 ± 0.028	
Alanine	0		0		0.085 ± 0.016	
Arginine	0.053 ± 0.009		0		0.018 ± 0.004	
Asparagine	n.d.		n.d.		0.012 ± 0.003	
Citrulline	0		0		0.044 ± 0.010	
Phenylalanine	0		0		0.132 ± 0.022	
Glycine	n.d.		n.d.		0.007 ± 0.002	
Glutamine	0.013 ± 0.008		0		0.038 ± 0.008	
Isoleucine	0		0		0.086 ± 0.016	
Histidine	0		0		0.014 ± 0.003	
Leucine	0.013 ± 0.008		0		0.217 ± 0.036	
Lysine	0		0		0.024 ± 0.005	
Methionine	0		0		0.054 ± 0.012	
Ornithine	n.d.		n.d.		0.01 ± 0.002	
Serine	n.d.		n.d.		0.039 ± 0.009	
Tyrosine	n.d.		n.d.		0.108 ± 0.019	
Threonine	n.d.		n.d.		0.026 ± 0.006	
Aspartic acid	n.d.		n.d.		0.081 ± 0.015	
Valine	0		0		0.085 ± 0.016	
**Total free amino acid**	0.079 ± 0.014		0 ± 0		1.248 ± 0.066	

* Calculated by determining the ratio of each essential amino acid per g of protein to the requirement as outlined by the WHO (2007). ** Tyrosine, while not an essential amino acid, is included in this category due to the requirement being a combined value with Phenylalanine.

**Table 3:** Functional properties of pea, soy and EverPro. Values in the same row that share a letter do not differ significantly.

	Pea	Soy	EverPro
pH	6.360 ± 0.030 ^a^	6.770 ± 0.010 ^b^	7.900 ± 0.000 ^c^
TTA (mL 0.1 M NaOH)	11.470 ± 0.020 ^a^	12.060 ± 0.260 ^a^	17.850 ± 0.100 ^b^
Protein solubility (%)	22.267 ± 1.457 ^a^	51.960 ± 3.354 ^b^	101.714 ± 2.898 ^c^
Surface Hydrophobicity (a.u.)	4292.467 ± 500 ^a^	7471.367 ± 324 ^b^	n.a.
Zeta potential at pH7 (mV)	−22.600 ± 1.633 ^a^	−33.778 ± 1.524 ^b^	−30.033 ± 2.035 ^b^
Separation rate (%/min)	1.327 ± 0.110 ^a^	0.990 ± 0.008 ^a^	4.135 ± 0.373 ^b^
Fat Absorption (%)	157.723 ± 3.202 ^a^	120.050 ± 17.847 ^b^	182.350 ± 1.909 ^a^
Colour L*	84.688 ± 1.164 ^a^	83.821 ± 0.603 ^a^	57.415 ± 2.379 ^b^
Colour a*	2.180 ± 0.036 ^a^	0.734 ± 0.049 ^b^	13.400 ± 0.455 ^c^
Colour b*	22.338 ± 0.215 ^a^	18.498 ± 0.129 ^b^	24.976 ± 0.804 ^c^

## Data Availability

The data are available from the corresponding author upon request.
